# A semi supervised approach to Arabic aspect category detection using Bert and teacher-student model

**DOI:** 10.7717/peerj-cs.1425

**Published:** 2023-06-08

**Authors:** Miada Almasri, Norah Al-Malki, Reem Alotaibi

**Affiliations:** 1 Information Technology Department/Faculty of Computing and Information Technology, King Abdulaziz University, Jeddah, Saudi Arabia; 2 European Languages Department/Faculty of Arts and Humanities, King Abdulaziz University, Jeddah, Saudi Arabia

**Keywords:** BERT, Transformer, AraBERT, Sentiment Analysis, Teacher model, Noisy Student model, Aspect Category Detection

## Abstract

Aspect-based sentiment analysis tasks are well researched in English. However, we find such research lacking in the context of the Arabic language, especially with reference to aspect category detection. Most of this research is focusing on supervised machine learning methods that require the use of large, labeled datasets. Therefore, the aim of this research is to implement a semi-supervised self-training approach which utilizes a noisy student framework to enhance the capability of a deep learning model, AraBERT v02. The objective is to perform aspect category detection on both the SemEval 2016 hotel review dataset and the Hotel Arabic-Reviews Dataset (HARD) 2016. The four-step framework firstly entails developing a teacher model that is trained on the aspect categories of the SemEval 2016 labeled dataset. Secondly, it generates pseudo labels for the unlabeled HARD dataset based on the teacher model. Thirdly, it creates a noisy student model that is trained on the combined datasets (∼1 million sentences). The aim is to minimize the combined cross entropy loss. Fourthly, an ensembling of both teacher and student models is carried out to enhance the performance of AraBERT. Findings indicate that the ensembled teacher-student model demonstrates a 0.3% improvement in its micro F1 over the initial noisy student implementation, both in predicting the Aspect Categories in the combined datasets. However, it has achieved a 1% increase over the micro F1 of the teacher model. These results outperform both baselines and other deep learning models discussed in the related literature.

## Introduction

Recent applications in business intelligence have considerably profited from access to beneficiaries’ online communication via various platforms (*e.g.*, social networks, forums, e-commerce sites, *etc*.). Those users’ views and opinions about numerous coarse or fine-grained services/products have impacted the design of such applications. Automatically analyzing this huge web of unstructured opinion data has been a practical research focus in recent years. Specifically, there is a surge in researchers’ work on improving machine learning (ML) and deep learning (DL) techniques used for sentiment analysis (SA) in business-oriented domains (Sun et al., 2019). In this research context, SA’s main objective is to discover opinions, extract the sentiments they represent, then classify these sentiments into polarities (Pang, Lee & Vaithyanathan, 2002). Recently, the focus of SA research has shifted towards enhancing sentiment polarity detection and classification. This, alongside the developments in the field of natural language processing (NLP), DL models, the emergence of aspect-based sentiment analysis (ABSA) have impacted current research in the field (Mowlaei, Saniee Abadeh & Keshavarz, 2020). ABSA’s sentiment classification is primarily conducted on aspect, sentence, or document levels. Generally speaking, the ABSA process involves aspect term extraction (ATE), aspect category detection (ACD), opinion term extraction (OTE), and aspect sentiment classification (ASC) (Mowlaei, Saniee Abadeh & Keshavarz, 2020).

As a sub-task of ABSA, ACD involves the categorization of a given sequence into a group of pre-defined aspect categories (Zhang et al., 2021). In business, detecting the aspect categories which demarcate the domain’s intelligence has recently become a priority research agenda. Particularly from a DL perspective, various supervised, unsupervised, and semi-supervised methods have been implemented to enhance the process of aspect extraction from reviews, forum content, social media, *etc*. These methods are implemented in practical business and marketing contexts like hotels and hospitality, tourism, dining and restaurants, e-commerce, gaming, *etc*. (Valdivia et al., 2020; Chauhan et al., 2020).

Implementing these DL methods for detecting coherent and representative aspects is always a challenging task for several reasons. Firstly, most of the current ACD research still focuses on testing supervised DL methods on labelled data. For example, datasets of user hotel reviews for which both aspect and SA polarity are defined. The research problem, in this case, is represented as a sequence labeling experiment which implements a sequence transduction model *e.g.*, long short-term memory (LSTM). Such solutions are basically context independent (Sun et al., 2020). Attention layers enhance long range dependencies in LSTMs and RNNs. Nonetheless, they do not support parallelization, subsequently making it inefficient in training and implementation. What is lacking in this research design is the attention to the overall semantic or contextual features of the data. These features are significant for defining the most representative aspect entities which could be utilized in an ABSA task. Therefore, what is needed are models which ensure that the contextual semantic domain is treated as input.

Secondly, reports on these supervised DL methods achieving best performance are understandably useful. Nonetheless, these reports are impractical when considering the cost of developing labeled data. Moreover, real-life user review data is unstructured, ever-changing, and unlabeled. This means it lacks a definitive identification of its main aspect entities (Dragoni, Federici & Rexha, 2019; Zhuang et al., 2020). In current ABSA research, the discussed DL solutions are primarily model-centric. These solutions seek to test and refine various implementations of models that proved their success or can be enhanced by the capabilities of other algorithms to achieve better performance (Yadav & Vishwakarma, 2020).

Data-centric (alternatively, customer-centric) designs are rarely used, especially in the context of ACD research. In such contexts, a small portion of the opinion data is available in labeled formats, whereas a larger component of this data is present as unlabeled. With the former type of data, supervised learning methods are implemented, whereas unsupervised learning is chosen in most cases with the latter. Alternatively, SSL or semi-supervised learning (that implements self-training) is different from unsupervised learning in that it can be applied to a range of problems such as clustering, regression, classification, and association. This differs from supervised learning which is reliant solely on labeled data, unlike SSL which utilizes both labeled and unlabeled data, thereby leading to less manual data annotation expenses and faster data preparation (Zhu, 2005; Van Engelen & Hoos, 2020).

To these challenges, one might add the challenge of researching ABSA, more specifically ACD, in a foreign language like Arabic. Arabic, for example, demonstrates a recognizable linguistic diversity and complexity as well as well-documented dialectical variation. This complexity is further observed in business-oriented contexts. In addition, research in Arabic ABSA has mainly applied methods that required extensive preprocessing and feature extraction steps. Moreover, researchers relied on external resources such as lexicons (Bensoltane & Zaki, 2021). Even when applying DL techniques, the focus was not on context-dependent applications. Instead, solutions like word embeddings (*e.g.*, word2vec), where each word has a fixed representation independent of its context, have been used (Abdelgwad, Soliman & Taloba, 2022).

Based on the previously discussed issues, this article aims to:

(1) Implement a semi-supervised learning (self-training) approach for aspect category detection (ACD) in Arabic hotel reviews, which utilizes both labeled (SemEval 2016) and unlabeled (HARD 2016) datasets. The former is smaller in comparison to the latter, thus encouraging the adoption of a data-centric approach.
(2) Use a four-step noisy student framework to improve the performance of a base Bert model (AraBERT) in an ACD task. This process involves training a teacher model on the labeled SemEval 2016 dataset, generating pseudo labels for HARD, training a noisy student on the combination of SemEval 2016 and HARD; finally, ensembling both teacher and student models to improve the performance of the proposed model.
(3) Evaluate the model on the combined datasets of labeled and unlabeled reviews using the micro F1 metric.

Thus, one can summarized the main contributions of this article as follows:

(a) To our knowledge, using BERT in a semi-supervised learning, self-training context to extract aspect categories from Arabic language hotel reviews has not been attempted.
(b) As with any semi-supervised DL experiment, we are seeking to use the aspects extracted from a labeled hotel review dataset (SemEval 2016) in a self-training learning context, to detect and classify the aspects in a larger unlabeled Arabic hotel review dataset (HARD 2016). This contributes as well to unexplored research problems where more emphasis is placed on the data and less attention is given to model complexity.
(c) To overcome the sequence dependence of some deep learning models, we use the transformer BERT (AraBERT v02) which is context-dependent, hence the contextual aspects of the reviews will be incorporated. This model overcomes the issues encountered with context-dependent solutions and even performs relatively better than attention enhanced models like RNNs and LSTMs. BERT facilities parallelization, which leads to efficient training and implementation.
(d) Benefiting from research done in the field of computer vision, the Teacher-student framework is used to enhance the BERT’s detection of aspect categories. To our knowledge, implementing this framework in Arabic ABSA or ACD research has not been attempted.

This study is motivated by its attempt to contribute to improving ACD accuracy in Arabic language settings through implementing a self-training approach that does not rely on vast sets of labeled data, unlike previous studies. This can help organizations and researchers in getting more accurate insights from Arabic textual data, improving the quality of products and services, and getting a better understanding of customers’ needs and preferences in Arabic-speaking regions.

The structure of the article is as follows. ‘Review of the Related Literature’ briefly reviews related work. Next, the experiments’ details and the proposed solution are explained in ‘Materials & Methods’. Results and findings are presented in ‘Results’. In ‘Discussion’, we discuss the major points of the findings. Section 6 highlights future research directions. Finally, we conclude the article in ‘Conclusions’.

## Review of the Related Literature

Early research in the field of SA has considered the overall polarity of sentences in review datasets. For example, a text polarity is perceived as representing one sentiment category, regardless of the internal entities to which this polarity might refer. Nonetheless, a sentence might include explicit or implicit references to more than one aspect: The sentence “This mobile’s 6.53-inch display is suitable to my needs, but its camera is its greatest failure” has two sentiment polarities. One sentiment is positive in relation to the display and the other is negative with reference to the camera.

The ABSA task is mainly composed of two interrelated sub-tasks. The first is aspect category detection (ACD) and the other is aspect category polarity (ACP). What is of interest to us in this research is the first sub-task. An ACD sub-task is basically a multi-label classification problem, where several review sentences, for example, are introduced as including a set of pre-defined aspect categories, *e.g.*, (Dining, Hygiene, *etc*.). Hence, the aim in such a task is to detect all the aspect categories which are presented in each sentence. Some of these aspect categories will be explicitly presented whereas others will be implicit. Ultimately, one review sentence can belong to one or more of the detected categories (Majumder et al., 2020; Xu et al., 2019; Do et al. , 2019).

Aspect extraction sentiment analysis detects different aspect terms in reviews. Topic modeling and latent Dirichlet allocation (LDA) were adopted for extracting product aspects from user reviews. The experimental results show that the proposed method is competitive in extracting product aspects from user reviews (Ozyurt & Akcayol, 2021). Considering Arabic sentiment analysis and mainly hotel reviews, authors in (Abdulwahhab et al., 2019) proposed an algorithm that analyzes the dataset using LDA to identify the aspects and their essential representative words to extract nouns and adjectives as possible aspects in the review.

Most recent DL research has indicated the improved performance of the developed language models when using transfer learning (TL), especially, in tasks related to SA and ABSA (Chen et al., 2017; Zhang, Wang & Liu, 2018). Basically, TL involves the process of pre-training a deep neural network (DNN) with weights learned from a different task where large, mostly, web-crawled data is being used. In a DL context, this entails reusing the weights of a pre-trained model in a new model while performing one of two tasks. One task entails retaining the fixity of model weights. The other involves fine-tuning those weights depending on the new task and data. Lower generalization errors and training time reduction are reported to be two of the major outcomes of implementing TL.

In ABSA research, TL has been implemented and resulted in improved language models. In (Fang & Tao, 2019), for example, the researchers propose a TL approach to solve a multi-label classification problem in the context of an ABSA task. They hypothesize that through using bidirectional encoder representations from transformers (BERT) to classify restaurant visitors’ opinions, they have been able to avoid additional preprocessing or transformation of their original data. The model achieved an accuracy of 61.65 in comparison to other tested approaches. In Tao & Fang (2020), the same researchers experimented with BERT and XLNet on different datasets: Restaurant, Wine, and Movies review datasets, and with an enhanced multi-hot encoding (reversible binary encoding). XLNet out-performed BERT on two datasets (restaurants = 66.65, and movies = 89.86), but generally, results of the experiments using BERT and XLNet have out-performed other DL techniques using LSTM, BiLSTM, CNN+LSTM plus other ML algorithms that have been used for baseline comparison.

With a specific focus on ACD, Ramezani, Rahimi & Allan (2020) have also used BERT with a supervised classifier to extract and classify 7 aspect categories from the OpoSum review dataset. Their base BERT model achieves high F1 on 5 of the identified categories in comparison to the other four models developed as baselines. Experimentation with TL in multi-lingual ABSA or ABSA-related tasks have also been reported to improve the model predictive performance. For instance, Winatmoko, Septiandri & Sutiono (2019) demonstrated the effectiveness of BERT and BERT+CRF models in OTE and ACD experiments, where BERT-CRF out-performed BERT on the Indonesian Airy Room hotel review dataset. Their models achieved an F1 of 0.92 and 0.93 on OTE and ACD respectively, which shows how enhancing context awareness with the CRF layer improved reflected positively on model performance. Similarly, Van Thin et al. (2021) pre-trained five BERT models (mBERT, mDistilBert, viBert4news, viBert FPT, and PhoBERT) on both mono-lingual and multilingual datasets from SemEval 2016 in the domains of hotels and restaurants. The researchers’ findings indicate that PhoBERT performed better compared to the other models on the mono-lingual datasets achieving an F1 of 86.53 on the restaurant dataset, and a 79.16 F1 on the hotel dataset. Similarly, researchers in Pathak et al. (2021) developed two BERT-based ensemble models (mBERT E MV and mBERT E AS) to detect aspects in user Hindi reviews and perform sentiment analysis through constructing auxiliary sentences from the detected aspects and feeding them into the network. They implemented these models on the labelled IIT-Patna Hindi dataset which covers topics related to Electronics, Mobile Apps, Travel, and Movies. Their results indicate that the mBERT E MV outperforms mBERT E AS and other three ML baselines (naive Bayes (NB), decision tree (DT), and sequential minimal optimization (SMO)) on the four sub-datasets.

Regarding ACD studies on the Arabic language, baseline research experimented with ML approaches to detect aspect categories in Arabic datasets. In Al-Smadi et al. (2016), for example, an SVM has been trained on the SemEval2016 Arabic dataset with special focus on tasks like aspect category detection (ACD), Opinion target expression (OTE), *etc*. The SVM achieved an F1 score of 40.3 on the ACD task. Researchers in Pontiki et al. (2016) also utilized ML techniques to perform ACD on the Arabic hotel reviews in the SemEval2016 dataset. The SVM implementation achieved an F1 score of 52.114.

ACD Arabic research that implemented DL techniques has started to demonstrate comparable results. Bensoltane & Zaki (2021) evaluated four types of word embedding approaches (AraVec, FastText, domain-specific, BERT) to enhance four models (LSTM, BiLSTM, GRU, BiGRU) using the SemEval 2016 Arabic hotel reviews dataset. The BERT embeddings achieved the highest F1 of 63.6 and 65.5 when added to the GRU and BiGRU models respectively. Kumar, Trueman & Cambria (2021) have proposed a convolutional stacked bidirectional LSTM with a multiplicative attention mechanism for aspect category detection and sentiment analysis. When evaluated, the CNN and stacked BiLSTM Multi Attn model achieved the same weighted F1 (=0.52) on the SemEval 2015 dataset, however it achieved a higher weighted F1 of 0.60 on the SemEval 2016 dataset than the CNN stacked BiLSTM Attn which scored 0.58. Similarly, researchers in Tamchyna & Veselovsk’a (2016) developed an LSTM network with a logistic regression layer to output a sentence classification as either positive (*i.e.,* including the E#A pair) or negative, which achieved the best F1 (=52.11) on the SemEval- 2016 hotel review dataset. Table 1 summarizes the main contributions of the related literature in the field of ACD either using ML or DL methods.

**Table 1:** Comparison of related literature.

**References**	**Dataset**	**Utilized model**	**Performance metrics**	**Evaluation results**	**Commentary**
Ozyurt & Akcayol (2021).	1,292 user reviews in Turkish language about smartphones.	Latent Dirichlet allocation (LDA) aspect-based-sentiment-analysis, sentence segment LDA (SS-LDA)	NA	results indicated that SS-LDA is successful in extracting products aspects from the reviews.	**Strengths**: LDA identifies patterns in text and groups words based on their similarity, making it fast and efficient for large datasets **Weaknesses**: Context-independent. Requires large datasets. Is not effective in identifying subtle aspects. Extracts only one topic per sentence
Abdulwahhab et al. (2019)	Seme-Eval 2015 dataset - hotel reviews	p_chunker algorithm for aspect extraction using latent Dirichlet analysis	precision, recall average, and F-score	The proposed p-chunker the precision was (0.79), recall average is (0.69), and F-score is (0.73)	**Strengths**: fast and efficient alternative for extracting latent topics **Weaknesses**: Context-independent. Does capture all aspects and might combine unrelated ones. Is not designed to handle complex sentence structures.
Chen et al. (2017)	-Movie review sentence polarity dataset v1.0 -Stanford sentiment treebank -Customer reviews of 5 digital products	BiLSTM CRF and CNN	Classification accuracy	82.3 88.3 85.4	**Strengths**: BiLSTM handles long-term dependencies and capture context information. CRF models dependencies between adjacent tokens and handle sequential patterns. CNN handles variable-length inputs **Weaknesses**: BiLSTM may suffer from vanishing gradient problems and can be slow to train. CRF is computationally expensive. CNN does not capture long-term dependencies.
Fang & Tao (2019)	restaurant reviews from Yelp.com (over 5 million reviews)	BERT	Accuracy, Hamming loss	Accuracy 61.65 Hamming loss 0.034	**Strengths**: BERT handles long-term dependencies, context awareness, and has adaptive word embeddings **Weakness**es: fine-tuning the model requires significant computational resources and may be slow
Tao & Fang (2020)	-Yelp -Wine Reviews -Rotten Tomatoes –Movie Reviews	BERT XLNet	Accuracy Macro F1 Micro F1	− 61.65, 0.48, 0.70 − 79.13, 0.86, 0.92 • 87.57, 0.95, 0.96	**Strengths**: Both BERT and XLNet handles long-term dependencies, context awareness, and has adaptive word embeddings **Weakness**es: fine-tuning both models require significant computational resources and may be slow
Ramezani, Rahimi & Allan (2020)	OpoSum dataset	Multi-layer perceptron (MLP) classifier	Micro-F1	56.5	**Strengths**: MLP handles both linear and non-linear relationships between features. **Weaknesses**: it is prone to overfitting and requires significant feature engineering
Winatmoko, Septiandri & Sutiono (2019)	Hotel reviews in ahasa Indonesia text	Transfer learning BERT-base and CRF	Micro F1	0.92	**Strengths**: CRF can model dependencies between adjacent tokens and handle sequential patterns, whereas BERT handles long-term dependencies, context awareness, and has adaptive word embeddings. **Weaknesses**: Both CRF and BERT computationally expensive
Van Thin et al. (2021)	-Vietnamese benchmark datasets -SemEval-2016	pre-trained BERT language models for the Vietnamese language	Micro F1 Precision Recall	86.53 85.60 87.49	**Strengths**: BERT handles long-term dependencies, context awareness, and has adaptive word embeddings **Weakness**es: fine-tuning the model requires significant computational resources and may be slow
Pathak et al. (2021)	IIT-Patna Hindi Reviews dataset for Indinal language (Electronics, Mobile Apps, Travel and Movies)	Ensemble models based on multilingual-BERT	Accuracy Micro F1 Precision Recall	Accuracy 70.49, 48.78, 75.47 and 79.77 in Electronics, Mobile Apps, Travel and Movies domains Micro F1 74.26, 59.70, 63.74 and 79.08 in the four respective domains.	**Strengths**: BERT handles long-term dependencies, context awareness, and has adaptive word embeddings **Weakness**es: fine-tuning the model requires significant computational resources and may be slow
Pontiki et al. (2016)	Arabic SemEval 2015 –Hotel reviews	SVM	Micro F1-score	52.114	**Strengths**: handles high-dimensional inputs and linear/nonlinear relationships between features. **Weaknesses**: Does not capture complex patterns well and requires significant feature engineering.
Al-Smadi et al. (2016)	Arabic SemEval 2015 –Hotel reviews	SVM	Micro F1-score	0.403	**Strengths**: handles high-dimensional inputs and linear/nonlinear relationships between features. **Weaknesses**: Does not capture complex patterns well and requires significant feature engineering.
Tamchyna & Veselovsk’a (2016)	SemEval 2016 –Hotel reviews	LSTM	Micro F1-score	0.473	**Strengths**: handles long-term dependencies and context information **Weaknesses**: may suffer from vanishing gradient problems and be slow to train.
Al-Smadi et al. (2018)	SemEval 2016 –Hotel reviews	RNN	Micro F1-score	0.480	**Strengths**: handles long-term dependencies and context information **Weaknesses**: may suffer from vanishing gradient problems and be slow to train.
Kumar, Trueman & Cambria (2021)	SemEval-2015 and SemEval-2016 dataset	CNN	Micro F1-score	0.52	**Strengths**: handles variable-length inputs. **Weaknesses**: does not capture long-term dependencies.
Bensoltane & Zaki (2021)	SemEval 2016	BERT embedding with BiGRU	Micro F1-score	0.655	**Strengths**: a combination of both models handle long-term dependencies and context information. **Weaknesses**: requires significant computational resources to train.

Despite the reported effectiveness of transfer learning, there has been a predominant concern about how it utilizes labels in the source task to learn network weights. This leads to bias, and to a less informative generalization on the target task because the classes which are found in the target labels are dissimilar to those in the source task (Yang et al., 2020). Therefore, researchers started experimenting with other less biased pretraining tasks like self-training, which is a wrapper method based on a semi-supervised learning approach. Initially, a classifier is trained on a small dataset of labeled instances then is used to classify a larger unlabeled dataset. The process involves adding the most confident predictions of the supervised model to the labeled dataset, then iteratively re-training it the model (Pavlinek & Podgorelec, 2017; Triguero, Garc’ıa & Herrera, 2015).

Though mainly achieving its state-of-the-art in image classification tasks, Self-training combined with pre-trained language models has demonstrated its usefulness in several text classification tasks (Du et al., 2020; Mukherjee & Awadallah, 2020). The sacristy of ABSA research harnessing the outcomes of self-training using the teacher-student framework is noted while conducting this review of literature; especially, in Arabic ACD. For example, researchers in Bhat, Sordoni & Mukherjee (2021) reported that a self-training approach with a teacher-student framework has been implemented on the ERASER benchmark dataset which encompasses five tasks. With specific focus on the SA task, they experimented with Base-Bert and achieved an F1 of 87 in a few-shot (25% training data) rationalization.

## Materials & Methods

Generally, the current research falls within the domain of “Subtask 1: Sentence level ABSA”, where researchers seek to identify an expressed opinion in documents regarding a target entity (*e.g.*, a TV, a store, or a hotel). More specifically, we engage with aspect category detection (ACD) where a pair of entities (laptop) and attribute (DESIGN) are to be detected. For this, we adapt the noisy student approach proposed and mostly researched in the field of computer vision.

### Datasets

To test the main assumptions of this research, two datasets have been used: the Arabic hotel reviews dataset from the SemEval 2016 and HARD, the Hotel Arabic-Reviews Dataset 2016. The first dataset is one of the contributions to the multilingual fifth task in the SemEval 2016 competition, which required the provision of customer reviews for ABSA in eight languages (Arabic, English, Chinese, Dutch, French, Russian, Spanish, and Turkish) sorted under seven topics (restaurants, laptops, mobile phones, digital cameras, hotels, museums, and telecommunications). The Arabic hotel reviews dataset is extracted from booking.com and tripadvisor.com, and is annotated, both on text level (2,291 texts) and sentence level (6,029 sentences).

**Figure 1:**
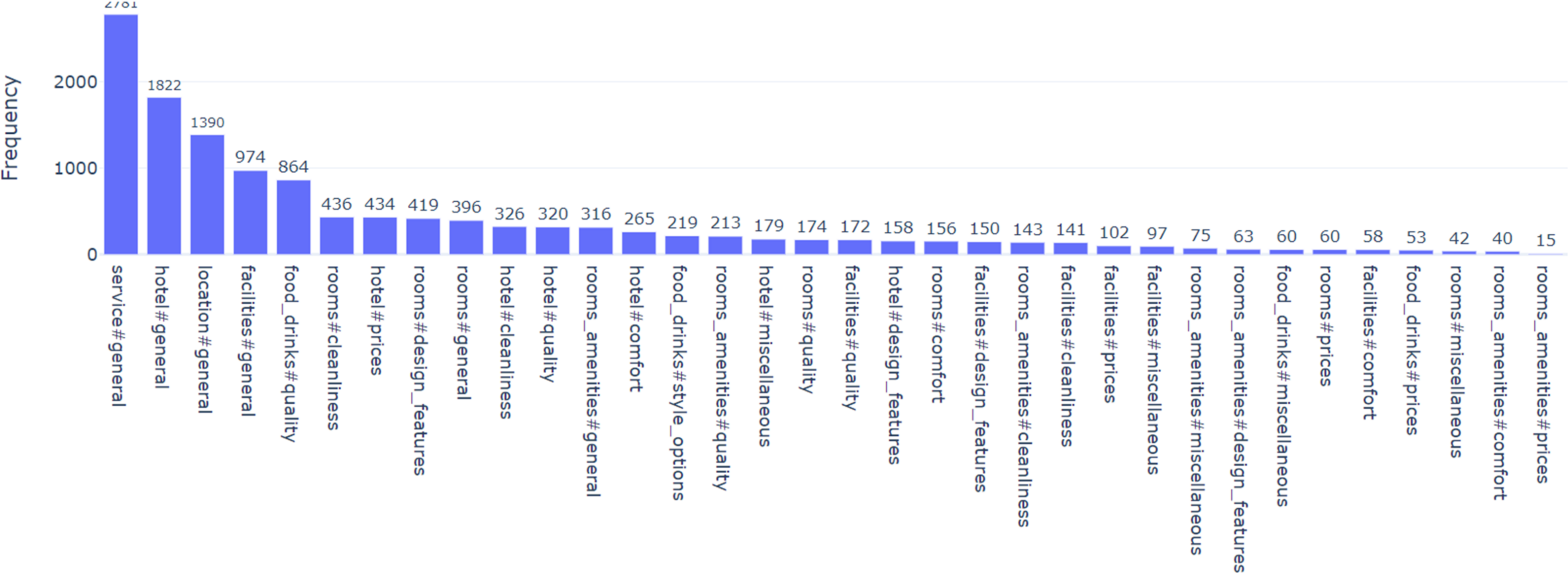
Distribution of some categories in SemEval2016 dataset.

For this dataset, aspects have been extracted, where an aspect category is decided based a pairing of Entity (hotel, rooms, facilities, room amenities, service, location, food drinks) and Attribute (general, price, comfort, cleanliness, quality, style options, design features, miscellaneous). Each sentence can be allocated to multiple categories resulting in a total of 34 categories. These categories are not distributed evenly in the dataset (Pontiki et al., 2016; Bensoltane & Zaki, 2021), and some of them are rare in the dataset as Fig. 1 shows.

**Figure 2:**
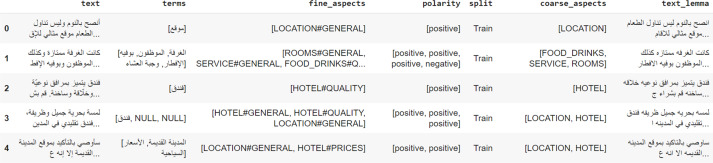
An example from the SemEval2016 dataset for hotel reviews.

We divided this dataset into a training set which includes 1,839 reviews, with a total of 4,802 sentences, and a Gold-standard Test Set. Figure 2 is an example from the SemEval 2016 dataset for hotel reviews. The test set contains 1,227 sentences extracted from 452 reviews as shown in Table 2.

**Table 2:** SemEval 2016 dataset.

Dataset	Train			Test		
	Texts	Sent.	Tuples	Texts	Sent.	Tuples
SemEval 2016 (Arabic)	1,839	4,802	8,757	452	1,227	2,158

**Figure 3:**
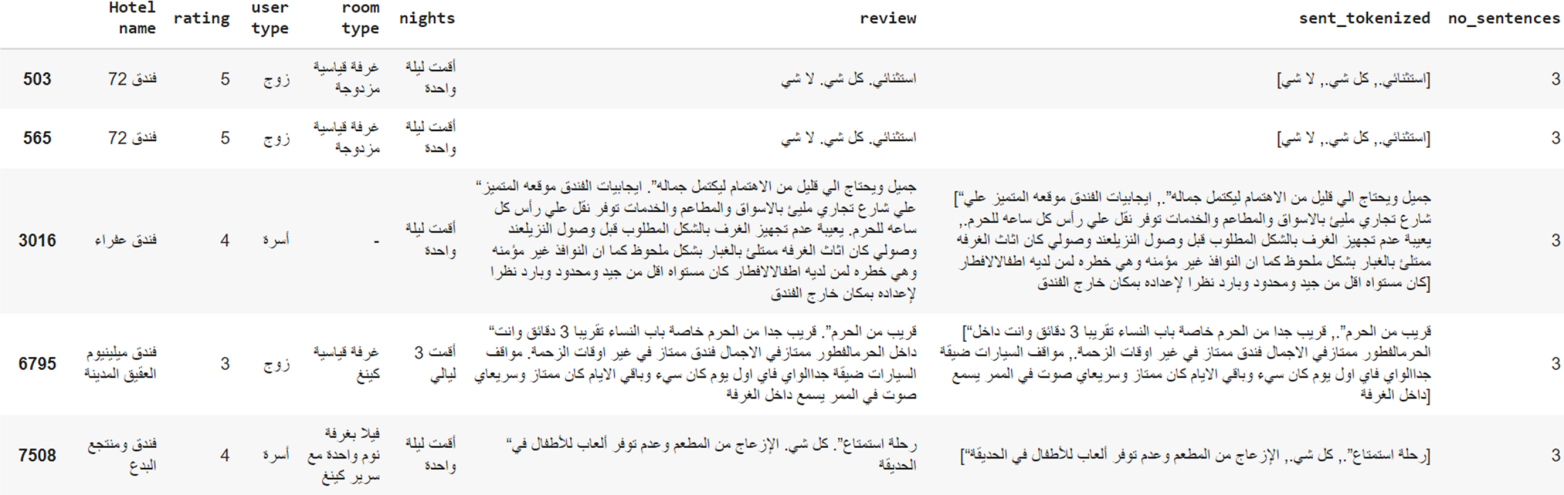
An example from the HARD dataset.

On the other hand, the HARD dataset is larger than the SemEval 2016 dataset and comprises 373,750 uncategorized hotel reviews about 1,858 hotels in a mix of standard and dialectal Arabic. This review dataset has been obtained from Booking.com in 2016 and consists of both balanced and unbalanced sets. Whereas the balanced dataset consists of 93,700 reviews and is equally divided into positive and negative records (46,850 each), the unbalanced one is made of the raw 373,750 reviews. The data is arranged in several columns: Hotel name, a rate (out of 5), user type (family, single, couple), room type, nights, title, positive review (+1), negative review (−1). The positive reviews make up 68% of the unbalanced HARD dataset, whereas the negative ones constitute 13% and the neutral 19%. Each record contains the review text in the Arabic language, and the reviewer’s rating on a scale of 1 to 5. In this research, the unbalanced dataset, which is made of review texts rated as either positive or negative, is used. Reviews with ratings 4 and 5 are mapped as positive, whereas reviews rated 1 and 2 are considered negative. The neutral rating is excluded. In its current state, the dataset is comprised of 93,700 reviews: 46,850 for each positive and negative classes (Elnagar, Khalifa & Einea, 2018). Figure 3 is an example from the HARD dataset.

### Preparation and pre-processing

Regarding the experiment set-up, it is conducted using python where Ktrain is utilized as a lightweight wrapper for our TensorFlow Keras implementation. Basically, the Ktrain library supports NLP experiments and working with text data in DL problems in supervised, semi-supervised or unsupervised learning tasks. Ktrain provides a learning rate finder, which can be used to discover an optimal learning rate for a model that is fitted to a specific dataset. Moreover, the Ktrain wrapper is useful in achieving improved generalizations and decreasing the model loss value through providing various types of learning rate schedules, for example, the triangular policy and Stochastic Gradient Descent With Restarts (SGDR).

Regarding the dataset, both datasets have undergone a uniform preprocessing step, where we have removed punctuation, normalized the words by dropping diacritics, replacing hamzated Alif with Alif, replacing AlifMaqsura with Yaa, normalizing Teh Marbuta, and removing Waaw at the beginning. As far as the HARD dataset, an alignment with the SemEval 2016 dataset is performed through segmenting the HARD full reviews into sentences, and removing extremely long sentences, so that the distribution of sentence lengths between the two datasets becomes similar.

### Noisy student to improve BERT classification

In this study, we implemented a semi-supervised learning approach that utilizes a noisy student training framework, which is a promising new technique that uses self-training and teacher-student learning to enhance the classification capability of AraBERT v02. The pretrained “AraBERT v02” (Antoun, Baly & Hajj, 2020) has been originally trained on about 8.2 billion words of MSA and dialectical Arabic. For the current implementation of the AraBERT v02, architecture and hyperparameters are set to be identical for both the teacher and student models in our experiments. The model consists of 12 hidden layers, 12 attention heads, and the hidden size of 768. After using the model to pre-process, tokenize, and prepare the dataset, the classifier layer in the AraBERT v02 is stored to perform sequence classification on a multilabel classification problem (Herrera et al., 2016).

As far as hyperparameters are concerned, the max sequence length has been set to be 86. The Adam optimizer is used to fine-tune the model. A triangular learning rate policy is being applied and set to the maximum learning rate of 1e−4 to reduce the demand for a constant tuning of the learning rate. The objective is to achieve the best classification accuracy results (Smith, 2017). The dropout rate is set to 0.1 with a hidden dropout probability of 0.3. Similarly, a batch size of 32 is applied, and the number of epochs is set to 10. Our noisy student framework adapts the methodology described in Xie et al. (2020).

(1) Simplified, a first step in this methodology involves the processing of the inputs from the labelled SemEval 2016 dataset to train a teacher model utilizing a standard cross entropy loss.
(2) The second step entails generating pseudo labels on the unlabeled HARD dataset using the previously developed teacher model. It is important to note that we did not add noise to the teacher model at this stage.
(3) In the third step of this process, a noised student model (that is equal in size to the previously developed teacher) is trained on the combined datasets with the objective of minimizing the combined cross entropy loss. Model noise is introduced into the student so that it does not only learn the teacher’s knowledge but goes beyond it. The noise parameters implemented are specifically dropout and stochastic depth function. We did not introduce noise into the data through augmentation, which is considered input noise. Using dropout removes dropout neurons from the neural network (AraBERT). As this is repeated for each training example, we end up with different models. The probabilities of each of those models are later averaged during the testing stage, which is similar to an ensembling step. Of significance to our research, and for purposes of comparison with the ImageNet 2012 used in Xie et al. (2020), where it is assumed that a balanced dataset has a positive effect on the performance of the student model, it must be observed that our unlabeled dataset, HARD 2016, is not balanced.
(4) Diverging from the fourth step described in Xie et al. (2020), which suggests an iteration process where student becomes teacher to generate pseudo labels on the unlabeled dataset, we opted for implementing an ensembling technique. The objective has been to utilize the moderately better performance of the student model in combination with the teacher. The input data of the combined datasets is run through both student and teacher models resulting in different probabilities, which are ultimately averaged.

**Figure 4:**
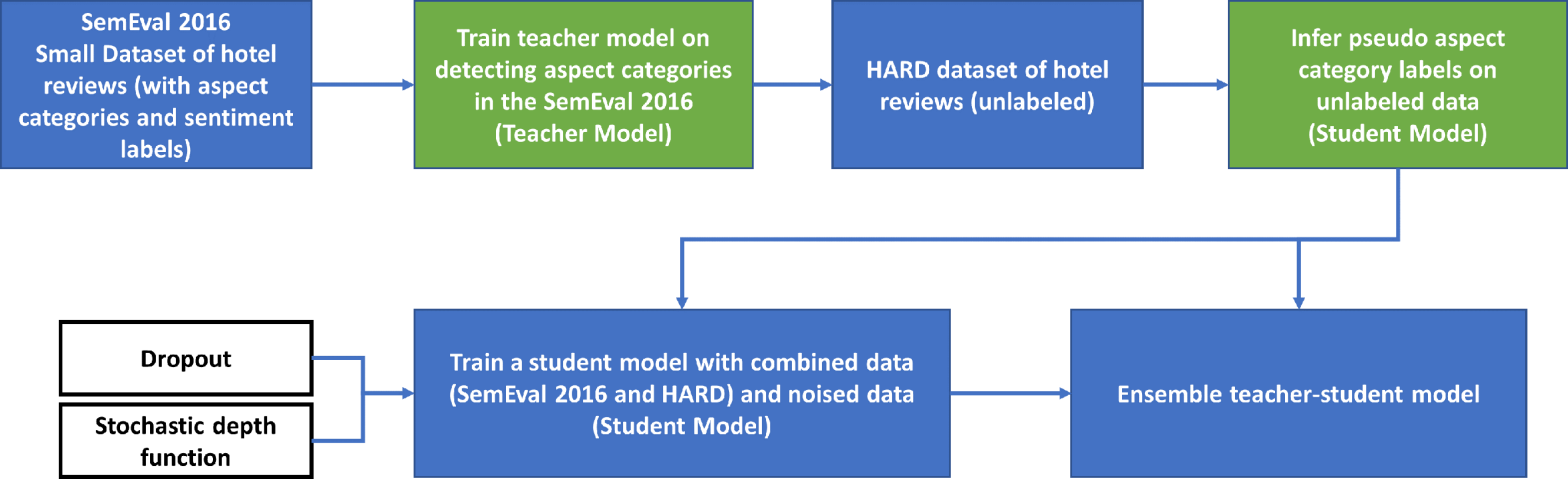
General workflow of the proposed solution.

This methodology will be implemented in four main steps. The general workflow of the proposed solution is shown in Fig. 4.

### Model evaluation

The Gold-standard Test Set, which is an unseen set extracted from the SemEval 2016 dataset has been used in the evaluation of the teacher, student, and ensemble implementations of our methodology. To evaluate our model, we used the Micro F1, which represents a micro-averaged F1, often employed to estimate the quality of multi-label binary problems. It computes the F1 of the combined inputs of all classes (Goutte & Gaussier, 2005) by summing the True Positives (TP), False Negatives (FN), and False Positives (FP), counting the instances for each, then inputting the results into the following equation:

\begin{eqnarray*}\text{Micro-F1}= \frac{\text{Precision Micro}\times \text{Recall Micro}}{\text{Precision Micro}+\text{Recall Micro}} \end{eqnarray*}

## Results

The results as demonstrated in Table 3 reveal a promising improvement in the performance of the AraBERT classification when implementing the noisy student framework. Generally, these results outperform baseline research and can be summarized as follows:

**Table 3:** Experimental results compared with related literature.

**Related Literature Baseline**	**Model**	**Precision**	**Recall**	**Micro F1 score**
Pontiki et al. (2016)	SVM	–	–	52.114
Al-Smadi et al. (2016)	SVM	–	–	0.403
Tamchyna & Veselovsk’a (2016)	LSTM	–	–	0.473
Al-Smadi et al. (2018)	RNN	–	–	0.480
Kumar, Trueman & Cambria (2021)	CNN	–	–	0.52
Bensoltane & Zaki (2021)	BERT embedding with BiGRU	–	–	0.655
**Proposed Model**	AraBert (Teacher)	0.75	0.58	0.672
	AraBert (noisy student on combined datasets)	0.72	0.64	0.679
	AraBert (Ensemble Teacher-Student)	0.72	0.64	**0.682**

(1) For the teacher model, which was trained on the labelled SemEval 2016 dataset, the micro F1 is 0.672 which initially indicates an improvement over reported baselines: the SVMs by 26.9%, the RNN and LSTM networks by 19.2%, the CNN by 15.1%, and the BERT (BiGRU) implementation by 1.7%.
(2) For the noisy student model which was trained on the combined dataset including both the SemEval 2016 and HARD 2016, the micro F1 is 0.679, which improves over the teacher implementation by 0.7% as well as demonstrates an improvement over the SVM-based baselines by 27.6%, the RNN and LSTM networks by 19.9%, the CNN by 15.8%, and the BERT (BiGRU) implementation by 2.4%.
(3) The ensemble teacher-student model which we trained on the combined dataset achieves a micro F1 of 0.682 which initially indicates an improvement over the noisy student by 0.03%, and by 1%. Moreover, the ensemble model outperforms the SVM-based baselines by 27.9%, the RNN and LSTM networks by 20.2%, the CNN by 16.1%, and the BERT (BiGRU) implementation by 2.7%. The previously cited results indicate that the ensemble model performs better in the ACD task compared to the teacher and student implementations.

## Discussion

Implementing a semi supervised self-training approach using a noisy student framework, as described in ‘Materials & Methods’ of this article, has led to recognizable improvement. The AraBERT classification results outperformed other models presented in related work on the SemEval 2016 hotel review dataset. A preliminary observation of the results confirms our assumption that the noisy student framework improves the performance of the AraBERT language model used in this study. The ensemble teacher-student model’s F1 score has achieved a 0.3% improvement when compared to the noisy student model. Similarly, ensemble teacher-student model’s F1 score has a 1% increase over that of the teacher model. As explored in detail in the findings, the proposed model with its ensemble teacher-student framework has considerably outperformed the baseline SVM-based models (Al-Smadi et al., 2016; Pontiki et al., 2016) by 27.9% in the micro F1. Compared to the RNN, LSTM, and CNN implementations reported in the related literature (Tamchyna & Veselovsk’a, 2016; Bensoltane & Zaki, 2021; Kumar, Trueman & Cambria, 2021), our model has demonstrated a significant improvement ranging from 16% to 20% in micro F1 metric. The proposed model has as well performed better than the highest performing DL model reported in the literature (BERT embedding with BiGRU) by 2.7% in the ACD task. We can attribute the progressive improvement in the various implementations of the framework to the significance of adding more data instances through combining the SemEval 2016 dataset with HARD dataset to create a large dataset which consisted of ∼ 1 million sentences. Moreover, with the noisy student implementation, we used a probability threshold to drop the examples of unlabeled data that the model has predicted with low confidence since these examples usually indicate out-of-domain aspects. The best results were obtained with a probability threshold of 0.4. This indicates that any example of the unlabeled data that the model did not predict with a probability of 0.4 or above, was dropped.

In addition, it was observed that the teacher model, though inferior to the student model, has performed better on some classes. Hence, the ensemble model was created from the predictions of the teacher and noisy student implementations to utilize this by-class difference. To achieve this, different weights have been tried, and the best result was obtained by giving each model the same weight of 0.5, which is equivalent to a simple mean.

**Table 4:** By-class aspect category detection results.

**Aspect category**	**Classes count**	**F1-score-(S)**	**SemEval**	**F1-score (T)**
ROOMS AMENITIES#PRICES	13	0	13	0
ROOMS#MISCELLANEOUS	30	0	30	0
ROOMS AMENITIES#COMFORT	35	0	33	0
FACILITIES#COMFORT	47	0	47	0
FOOD DRINKS#MISCELLANEOUS	51	0	51	0
ROOMS AMENITIES#DESIGN FEATURES	54	0	54	0
ROOMS AMENITIES#MISCELLANEOUS	55	0	55	0
FACILITIES#MISCELLANEOUS	70	0	70	0
FACILITIES#QUALITY	159	0	136	0
FOOD DRINKS#PRICES	195	0	41	0
ROOMS#PRICES	505	0	46	0.2353
ROOMS#QUALITY	898	0.0645	142	0.1212
HOTEL#MISCELLANEOUS	1,156	0	140	0
HOTEL#DESIGN FEATURES	3,982	0.4103	127	0.4186
FACILITIES#PRICES	4,633	0.1	84	0.2727
FACILITIES#CLEANLINESS	7,566	0.3684	115	0.2222
ROOMS#GENERAL	7,880	0.541	319	0.5378
FACILITIES#DESIGN FEATURES	8,542	0.1667	117	0.0625
ROOMS AMENITIES#CLEANLINESS	9,405	0.24	123	0.1905
ROOMS AMENITIES#GENERAL	13,817	0.3373	251	0.2535
ROOMS#COMFORT	13,847	0.5333	135	0.5
FOOD DRINKS#STYLE OPTIONS	15,080	0.6667	182	0.5588
HOTEL#QUALITY	15,547	0.2921	253	0.3478
ROOMS AMENITIES#QUALITY	23,045	0.3137	176	0.3556
HOTEL#COMFORT	33,800	0.4889	199	0.5684
ROOMS#DESIGN FEATURES	38,392	0.7068	342	0.6557
ROOMS#CLEANLINESS	41,187	0.7574	357	0.7417
FOOD DRINKS#QUALITY	47,833	0.8408	686	0.8339
HOTEL#PRICES	54,350	0.6438	372	0.6324
HOTEL#CLEANLINESS	74,409	0.6525	252	0.5882
FACILITIES#GENERAL	123,390	0.5114	785	0.5163
LOCATION#GENERAL	177,198	0.8342	1,087	0.8464
SERVICE#GENERAL	218,753	0.9146	2,221	0.9018
HOTEL#GENERAL	417,055	0.7198	1,468	0.7074

**Figure 5:**
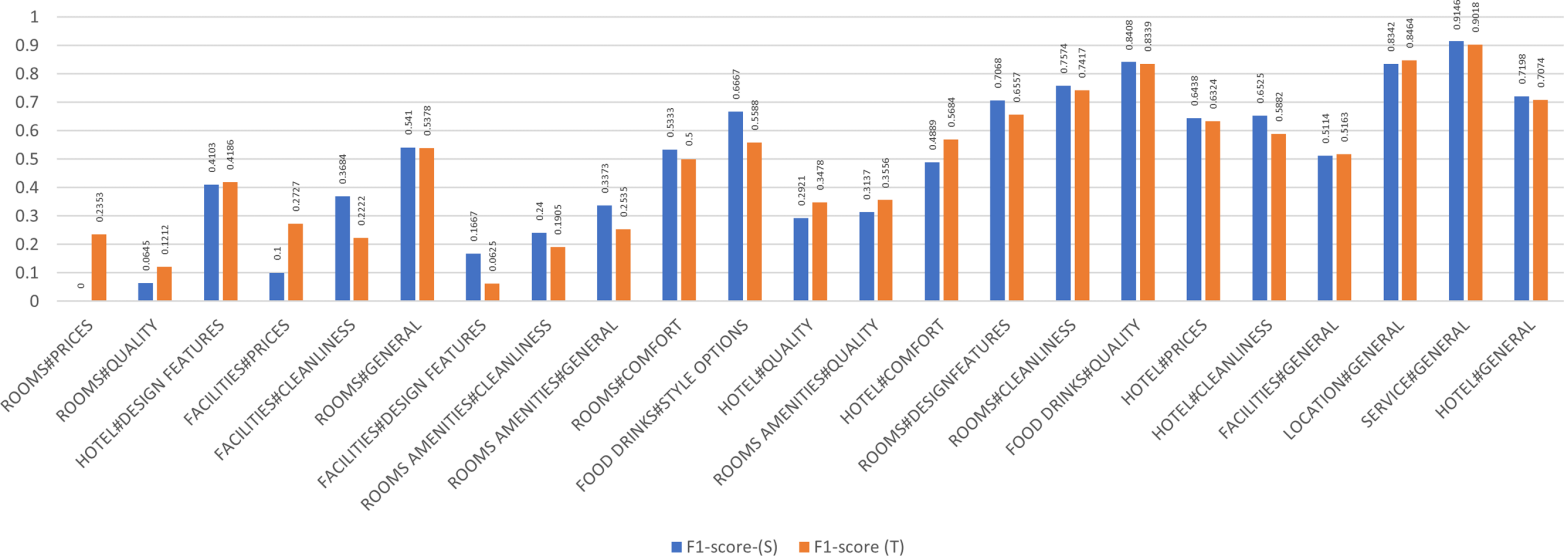
By aspect micro F1 (Teacher-Student).

A detailed examination of the aspects as shown in Fig. 5 reveals that out of the 34 aspects, the teacher detected 23 aspects, however failed to identify 11, whereas the student identified 22 and failed to detect 12. The inability of the model to detect those aspects is merely attributed to their limited representation in the datasets. For example, and according to Table 4 the category count of the rooms amenities#prices aspect in the datasets is 13 in both SemEval 2016 and combined datasets, hence getting an F1 of 0.00 due to the limited number of aspects. The service#general category achieves a 0.91 and a 0.90 F1 in the Teacher, and Student models because the category count in both implementations is 218,753 and 2,221 respectively, indicative of a higher representation of classes.

## Conclusions

This study utilizes the Arabic version of the BERT model, AraBERT v02, to conduct a semi-supervised self-training approach using a noisy student framework to perform an ACD task on two datasets: a labelled and unlabeled hotel review datasets (SemEval 2016, and HARD 2016). Experimental findings revealed that an ensembled teacher-student model achieves the best F1 (0.68), hence outperforming the baseline and related work on the same dataset. The by-class F1 are relatively affected by the size of category representation in the datasets. Our future work in ACD research targets experimenting with other methods to introduce noise into the student model like augmentation and conducting student iterations. Moreover, and while adopting the same data-centric perspective, we seek to enhance the quality of the data being used through feature engineering and dealing with dialectical and lexical challenges in the Arabic language. It is expected that further enhancements of both data quality and the noisy student framework might significantly improve AraBERT text classification capability to outperform existing models in the ACD task on the Arabic language. Future work on this project might entail increasing the size of the unlabeled dataset, performing several student iterations, and experimenting with more complex BERT architecture, while implementing the noisy student framework might prove effective in future research experiments.

## Additional Information and Declarations

### Data Availability

The following information was supplied regarding data availability: The HARD dataset is available at GitHub: https://github.com/elnagara/HARD-Arabic-Dataset. Elnagar A., Khalifa Y.S., Einea A. (2018) Hotel Arabic-Reviews Dataset Construction for Sentiment Analysis Applications. In: Shaalan K., Hassanien A., Tolba F. (eds) Intelligent Natural Language Processing: Trends and Applications. Studies in Computational Intelligence, vol 740, pp: 35-52. Springer International Publishing. doi= 10.1007/978-3-319-67056-0_3. url=10.1007/978-3-319-67056-0_3
The SemEval2016 dataset is available at GitHub: https://github.com/msmadi/ABSA-Hotels. Mohammad, A. S., Qwasmeh, O., Talafha, B., Al-Ayyoub, M., Jararweh, Y., & Benkhelifa, E. (2016, December). An enhanced framework for aspect-based sentiment analysis of Hotels’ reviews: Arabic reviews case study. In 2016 11th International Conference for Internet Technology and Secured Transactions (ICITST) (pp. 98-103). IEEE. Al-Smadi, M., Talafha, B., Al-Ayyoub, M., & Jararweh, Y. (2019). Using long short-term memory deep neural networks for aspect-based sentiment analysis of Arabic reviews. International Journal of Machine Learning and Cybernetics, 10(8), 2163-2175. Pontiki, M., Galanis, D., Papageorgiou, H., Androutsopoulos, I., Manandhar, S., Mohammad, A. S., ... & Hoste, V. (2016, June). Semeval-2016 task 5: Aspect based sentiment analysis. In Proceedings of the 10th international workshop on semantic evaluation (SemEval-2016) (pp. 19-30). The code is available at GitHub and Zenodo:
https://github.com/Malmasre/Arabic-ACD; Malmasre. (2023). Malmasre/Arabic-ACD: Arabic-ACD (Arabic). Zenodo. https://doi.org/10.5281/zenodo.7803382.
